# Supplementary material for: Tenascin-C activation of lung fibroblasts in a 3D synthetic lung extracellular matrix mimic
Source: bioRxiv. 2023 Feb 24:2023.02.24.529926. Preprint. [Version 1] doi: 10.1101/2023.02.24.529926 (PMC9980292; doi:10.1101/2023.02.24.529926)
Supplement: Supplementary file 1 [file NIHPP2023.02.24.529926v1-supplement-1.pdf]

bioRxiv preprint doi: https://doi.org/10.1101/2023.02.24.529926; this version posted February 24, 2023. The copyright holder for this preprint (which was not certified by peer review) is the author/funder, who has granted bioRxiv a license to display the preprint in perpetuity. It is made available under aCC-BY 4.0 International license.

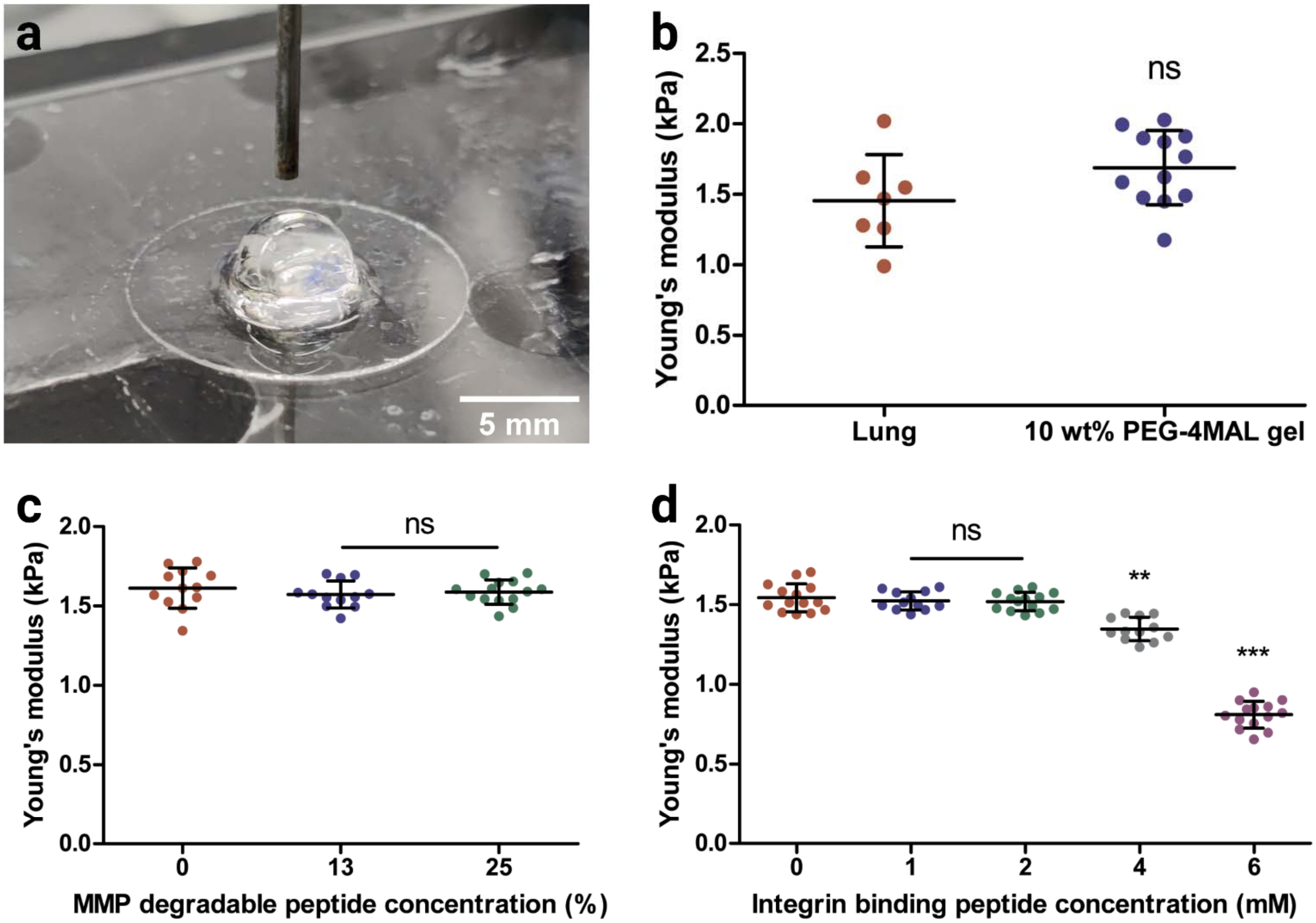

Supplementary Figure 1. Mechanical characterization of lung hydrogels using micro-indentation method. a) Representative image of lung hydrogel after 24 hours of swelling in 1X PBS. Modulus of the hydrogel was measured using micro-indentation method with a 1 mm diameter flat probe. b) Effective modulus of 10 wt% 4-arm PEG-maleimide hydrogel without bioactive peptide ligands using micro-indentation replicated the lung modulus measured using the same method. c) Effective modulus of 10 wt% 4-arm PEG-maleimide hydrogel containing different molar concentrations of MMP-degradable peptide crosslinkers using micro-indentation method. The tested peptide crosslinker molar concentrations were 0%, 13% and 25%. d) Effective modulus of 10 wt% 4-arm PEG-maleimide hydrogel containing different molar concentrations of integrin-binding peptide moieties using micro-indentation method. The tested peptide molar concentrations were 0 mM, 1 mM, 2 mM, 4 mM, and 6 mM.

bioRxiv preprint doi: https://doi.org/10.1101/2023.02.24.529926; this version posted February 24, 2023. The copyright holder for this preprint (which was not certified by peer review) is the author/funder, who has granted bioRxiv a license to display the preprint in perpetuity. It is made available under aCC-BY 4.0 International license.

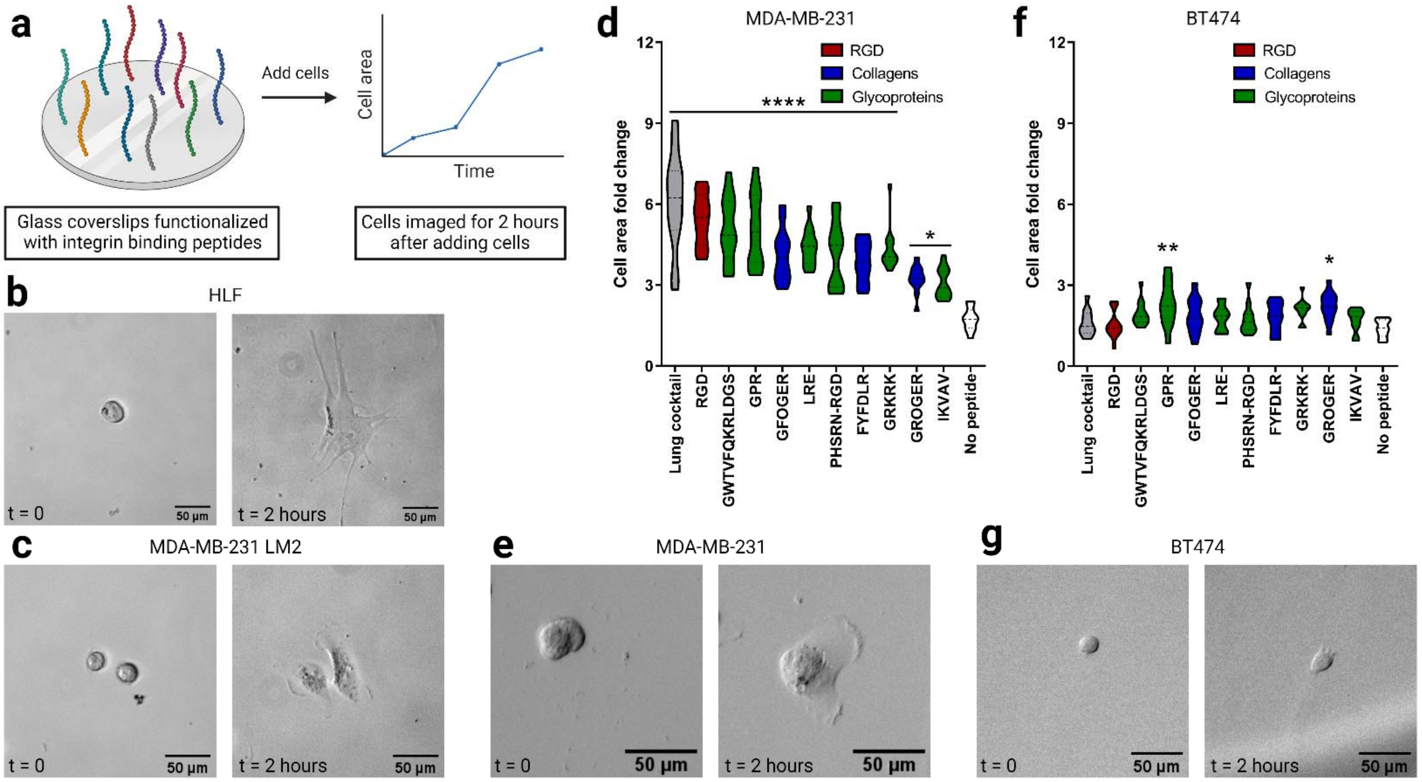

Supplementary Figure 2. Functional validation of lung integrin-binding peptides. a) Schematics of the cell attachment assay where cells were seeded on glass coverslips functionalized with lung integrin-binding peptides and the cells were imaged for 2 hours. b) Representative images of HLFs at time t = 0 and t = 2 hours. c) Representative images of MDA-MB-231 LM2 cells at time t = 0 and t = 2 hours. d) Cell area fold change for MDA-MB-231 cells in compared to negative control. e) Representative images of MDA-MB-231 cells at time t = 0 and t = 2 hours. f) Cell area fold change for BT474 cells in compared to negative control. g) Representative images of BT474 cells at time t = 0 and t = 2 hours.

bioRxiv preprint doi: https://doi.org/10.1101/2023.02.24.529926; this version posted February 24, 2023. The copyright holder for this preprint (which was not certified by peer review) is the author/funder, who has granted bioRxiv a license to display the preprint in perpetuity. It is made available under aCC-BY 4.0 International license.

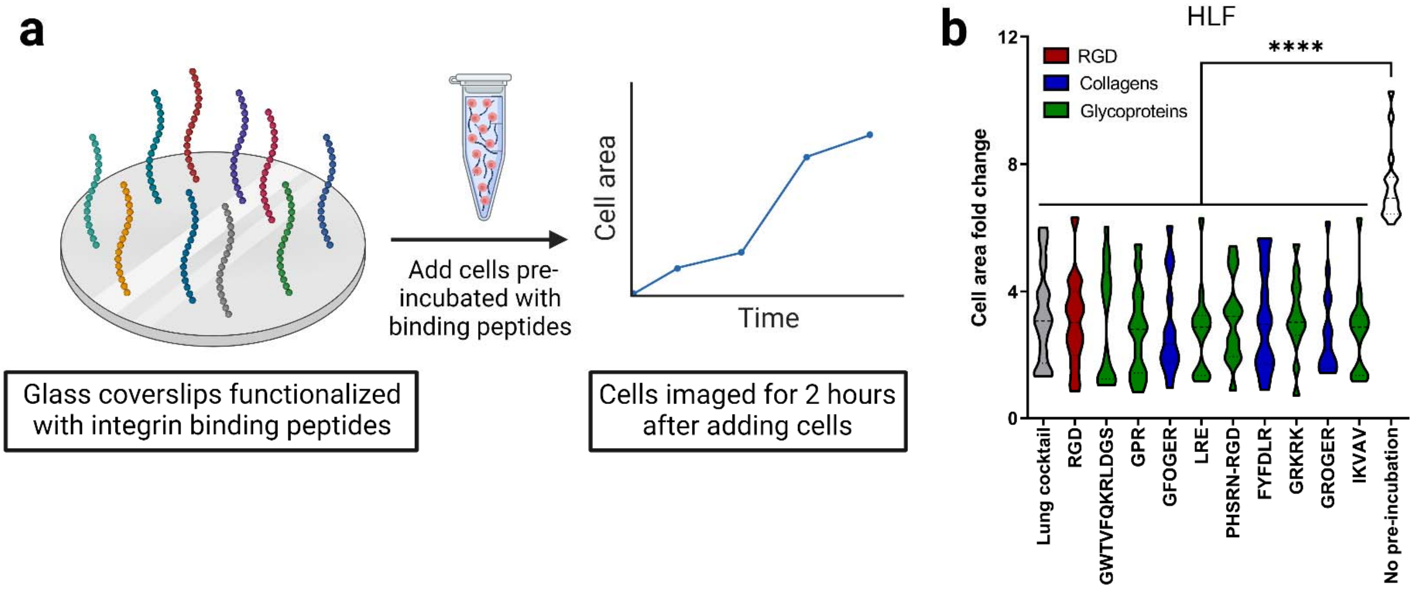

Supplementary Figure 3. Competitive cell attachment assay on lung integrin-binding peptides using HLFs. a) Schematics of the competitive cell binding assay where HLFs pre-incubated with individual lung integrin-binding peptides were seeded on glass coverslips functionalized with lung integrin-binding peptide cocktail and the cells were imaged for 2 hours. b) Representative images of HLFs (pre-incubated with individual binding peptides) at time t = 0 and t = 2 hours.

bioRxiv preprint doi: https://doi.org/10.1101/2023.02.24.529926; this version posted February 24, 2023. The copyright holder for this preprint (which was not certified by peer review) is the author/funder, who has granted bioRxiv a license to display the preprint in perpetuity. It is made available under aCC-BY 4.0 International license.

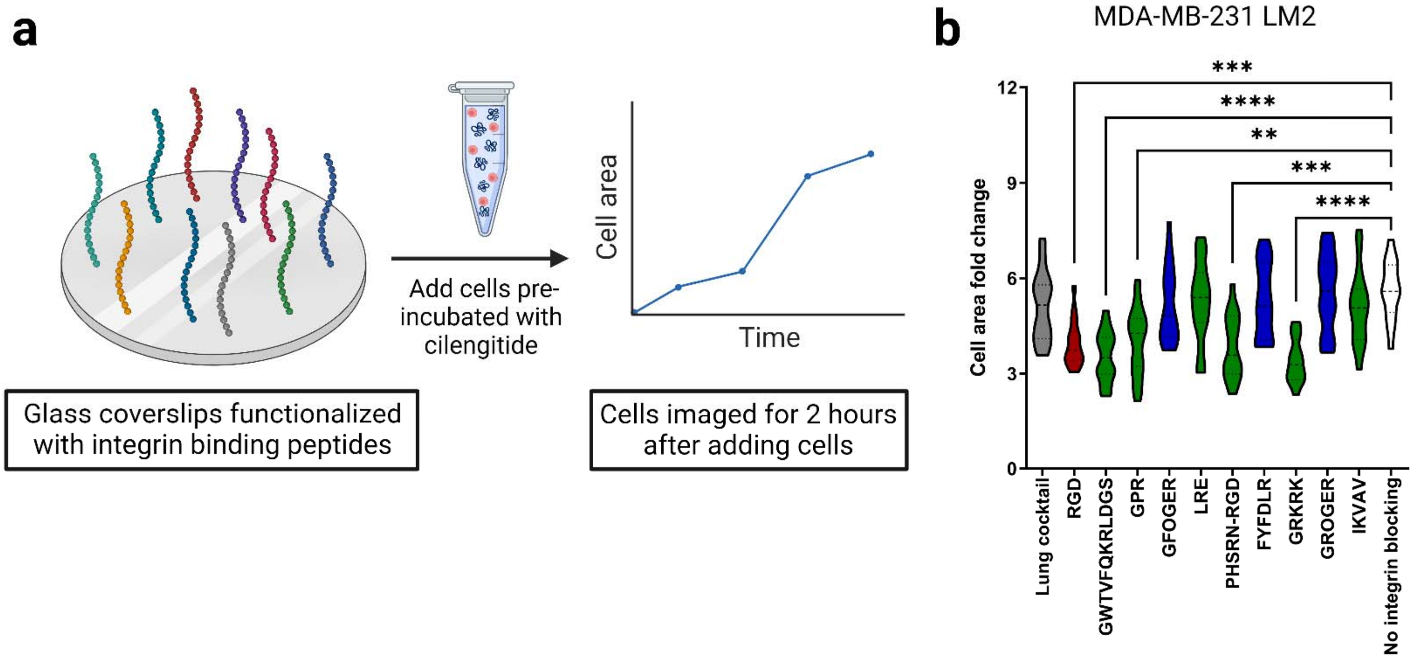

Supplementary Figure 4. Integrin-specific cell attachment with lung integrin-binding peptides. a) Schematics of the competitive cell binding assay where HLFs pre-incubated with cilengitide were seeded on glass coverslips functionalized with lung integrin-binding peptide cocktail and the cells were imaged for 2 hours. b) Cell area fold change 2 hours after seeding HLFs (pre-incubated with cilengitide) onto glass coverslips functionalized with integrin-binding peptide cocktail relative to a negative control (cells not pre-incubated with cilengitide).

bioRxiv preprint doi: https://doi.org/10.1101/2023.02.24.529926; this version posted February 24, 2023. The copyright holder for this preprint (which was not certified by peer review) is the author/funder, who has granted bioRxiv a license to display the preprint in perpetuity. It is made available under aCC-BY 4.0 International license.

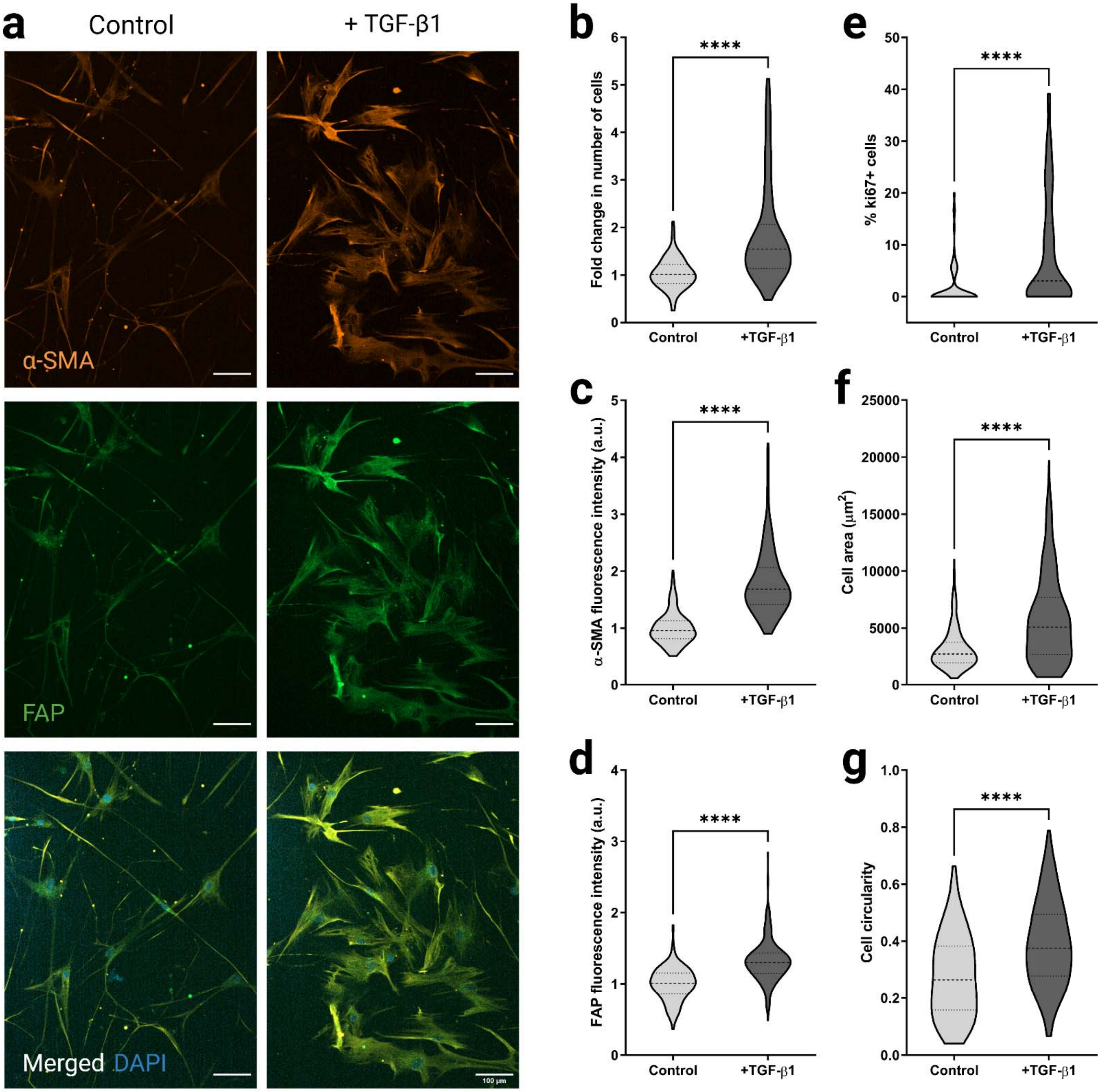

Supplementary Figure 5. Fibroblast phenotype and activation on TCPS. (a) Representative fluorescent images of HLFs cultured on TCPS with or without pro-fibrotic cytokine, TGF-β1 showing a-SMA (orange), and FAP (green) expressions along with merged HLF images with nuclei staining with DAPI (blue). (b) Fold change in cell count for non-activated (cultured without TGF-β1) and activated (cultured with TGF-β1) HLFs on TCPS representing cell proliferation characteristics. (c) Quantification of a-SMA expression from non-activated and activated HLFs cultured on TCPS. (d) Quantification of FAP expression from non-activated and activated HLFs cultured on TCPS. (e) Percentage of proliferative ki67+ cells in non-activated and activated HLF cultures on TCPS. (f) Cell area for non-activated and activated HLFs cultured on TCPS. (g) Cell circularity for non-activated and activated HLFs cultured on TCPS.

bioRxiv preprint doi: https://doi.org/10.1101/2023.02.24.529926; this version posted February 24, 2023. The copyright holder for this preprint (which was not certified by peer review) is the author/funder, who has granted bioRxiv a license to display the preprint in perpetuity. It is made available under aCC-BY 4.0 International license.

a

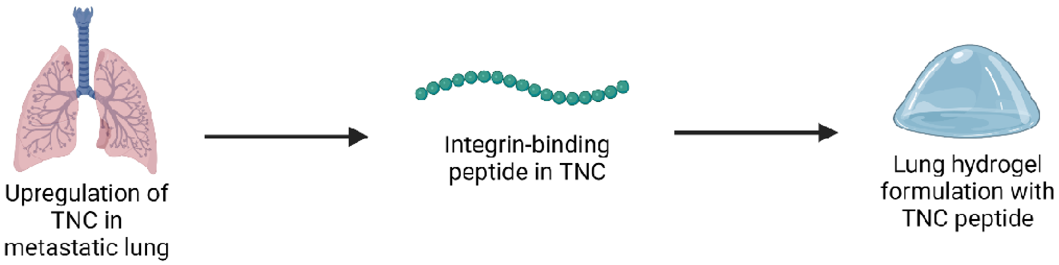

b **ECM proteins** with known **integrin-binding domains**

Collagen VI (α1, α2, and α3), Emilin I, Fibrillin I, Fibrinogen β, Nidogen I, Nephronectin, Vitronectin, von Willebrand Factor	Fibrinogen α	Collagen IV (α1, α2)
	**GPR**GGC, 8%	GCG**FYFDLR**, 4%
	Collagen I (α1, α2)	Elastin
G**RGD**SPCG, 32%	CGP(GPP)$_5$**GFOGER**(GPP)$_5$, 6%	GC**GRKRK**, 2%
Tenascin C	Laminin γ	Collagen III (α1)
CGG**AEIDGIEL**, 22%	GCK**QLRE**Q, 6%	CGP(GPP)$_5$**GROGER**(GPP)$_5$, 2%
Fibrinogen γ	Fibronectin	Laminin α
G**CGWTVFQKRLD**GS, 10%	CG**PHSRN**G$_6$**RGD**S, 5%	CSRARKQAAS**IKVAV**ADR, 2%

c Synthesize **peptides** degradable by target **MMPs**

MMP1	MMP2
GCRD**VPMS/MRGG**DRCG, 16%	GCRD**SGESPAY/YTA**DRCG, 16%
MMP3	MMP7
GCRD**RPFS/MIMG**DRCG, 16%	GCRD**VPLS/LTMG**DRCG, 16%
MMP9	MMP13
GCRD**VPLS/LYSG**DRCG, 12%	GCRD**GPLG/LWAR**DRCG, 13%
MMP14	
GCRD**IPES/LRAG**DRCG, 12%	

Supplementary Figure 6. List and quantities of metastatic lung-specific integrin-binding and MMP-degradable peptides. a) Schematic of identification of metastatic lung ECM-specific integrin-binding and MMP-degradable peptides. b) List of identified integrin-binding peptide domains with their relative quantities corresponding to metastatic lung ECM proteins. c) List of identified MMP-degradable peptide domains with their relative quantities corresponding to the different MMPs.
